# 

**Published:** 1906-08-15

**Authors:** 


### CONTENTS.

Event and Comment - - - - - - - - - - 379

Proving the Articulation, Contour and Expression, By George H. Wilson, D.D.S. 382

Why the So-called Gold Annealer Should be Used in Giving the Heat Treatment to Gold Foil, - By M. L. Ward, D.D.S. 388

Controling a Hypersensitive Palate When Taking Impressions, By A. E. Franklin, D.D.S. 398

Dentistry a Worthy Profession, - - By Prof. S. H. Guilford 399

Oral Hygiene A Necessity to Health, By Burton Lee Thorpe, M.D., D.D.S. 403

With Our Editors - - - - - - - - - 405

Indents - - - - - - - - - - - - 412

Announcements - - - - - - - - - - 430

